# Kademlia hash snow ablation resource optimized stride scheduling for mobile computing services in healthcare sector

**DOI:** 10.1038/s41598-025-15913-w

**Published:** 2025-11-28

**Authors:** Nithya Rekha Sivakumar

**Affiliations:** Department of Computer Sciences, College of Computer and Information Sciences, Princess Nourah bint Abdulrahman University (PNU), P.O. Box 84428, Riyadh, 11671 Saudi Arabia

**Keywords:** Internet of things, Mobile computing, Artificial intelligence, Kademlia hash function, Snow ablation optimization, Stride scheduling, Health care, Engineering, Mathematics and computing

## Abstract

Healthcare plays an essential role in every individual’s life. Smart healthcare employs a new generation of information technologies like Internet of Things (IoT), Mobile Computing (MC), and Artificial Intelligence (AI) to transform the conventional medical system into a modernized one. Many researchers have reached better healthcare solutions by ensuring robust and versatile accessibility to the people. Healthcare applications on mobile devices exchange data through communication interfaces between patients and healthcare service providers. However, every patient’s data possess a significant amount of computing resources like Central Processing Unit (CPU), network bandwidth, and memory, and hence cannot be processed and validated at the same time. In order to overcome resource limitations and make scheduling efficient, mobile devices contain patient data that is integrated with the scheduling process to provide the resource efficient mobile computing services in the healthcare sector. Kademlia HashSnow Ablation Resource Optimized Stride Scheduling (KHSAROSS) is proposed in mobile computing. The novelty of the proposed KHSAROSS method is designed to build mobile phone based remote healthcare monitoring to identify the resource optimized virtual machine and balance the overloaded virtual machine. The key advantage of the proposed KHSAROSS method is to enhance the mobile computing services in the healthcare sector while increasing makespan. The KHSAROSS method involves three distinct processes, namely the patient data collection task, optimization and scheduling. Sensors with a data acquisition unit are used to acquire patients’ physiological states like temperature, heart rate, and Electroencephalography (EEG) data to perform mobile phone based remote healthcare monitoring. The Kademlia Hash Function here generates a hash value for each patient’s collected data. Following this, Snow Ablation Optimization is carried out to identify resource optimized (i.e., CPU, network bandwidth, and memory) virtual machines (i.e., healthcare service providers) for performing scheduling of stored patient data. Finally, Stride Scheduling is used to balance overloaded virtual machines with less loaded virtual machines. This, in turn, ensures efficient resource allocation and scheduling in mobile computing. The effectiveness of the proposed and existing methods is assessed using metrics for scheduling accuracy, scheduling time, throughput and makespan.

## Introduction

Over the past few years, with the full amalgamation and application of state-of-the-art technologies, to name a few, artificial intelligence and cloud computing, the Internet of Healthcare Things (IoHT), a paramount application area of IoT technology in the healthcare industry, has progressively shown strong influence and vitality. Nevertheless, there are numerous resource concerns, to name a few being battery life, bandwidth, and storage space issues unique to mobile devices. As a result, the service quality is said to be heavily influenced by these issues.

A computational task offloading method called Online Offloading based on the Deep Reinforcement Learning (OO-DRL) in smart healthcare networks with non-divisible features and a tradeoff between latency and energy consumption to converge the necessities of high-reliability services was proposed in^1^. The OO-DRL offloading algorithm via parallel deep neural networks and a weight-sensitive experience replay mechanism was designed to minimize real-time task processing latency and energy consumption extensively. However, the accuracy and time involved in the process of scheduling were not focused. To concentrate on this aspect, a Snow Ablation-based Resource Optimization and Stride Scheduling model is designed to improve the overall accuracy and reduce time.

Owing to constrained resources, mobile devices frequently make use of cloud computing offloading characteristics to perform more complicated tasks. In^2^, a non-dominated multi-objective strategy taking into consideration the Harris Hawks Optimization (HHO), referred to as the Hybrid Multi-objective Harris Hawks Optimization (HMHHO), was proposed with the intent of handling issues concerning constrained resources. With this type of design, the HMHHO method not only accomplished jobs quickly but also minimized energy significantly. In spite of improvements in energy, the latency and makespan involved for mobile computing services in the healthcare sector have not been addressed. To concentrate on these issues, the Kademlia Hash Function is employed, which generates a hash value for each patient’s collected data, therefore considering overall latency and makespan.

Aging populations, the mutation of critical illnesses to chronic diseases, and economic coherences related to the transfer of patient management to the home, instead of specialized institutions, are steering the application of internet-enabled home care mechanisms and specifically remote health monitoring along with mobile health solutions. A holistic exploration of the integration of Artificial Intelligence (AI) into healthcare, concentrating on its transformative inferences and challenges, was investigated in^3^. The employment of machine learning techniques in health care is swiftly improving. Nevertheless, the procedures that aid these tools in deployment employing machine learning operations are still in the infant stage. In^4^, not only holistic fusions of prevailing in the field of healthcare but also the gaps were identified, and insights for adoption in clinical practice were presented.

A transient overview of AI along with its contributions and real-world applications in the healthcare domain was investigated in^5^. Also, its influence on medical education, along with the ethical issues of its integration into the medical field was discussed in depth. However, issues concerning user capacity, network speed, price, and resources involved were not focused. To concentrate on these aspects, several research issues for 5G-based smart healthcare were discussed in^6^. Nevertheless, any downtime, in the case of critical health services, could result in patient health issues and also cause mortality. In^7^, an interdisciplinary mechanism integrating stochastic methods with optimization techniques was presented with the intent of analyzing how failures influence e-health monitoring system accessibility.

With the evolution of IoT, E-healthcare applications are making an appearance and reaping popularity. These E-healthcare applications necessitate low latency and rigorous computation or storage resources. But, IoT terminals possess constrained resources that are found to be inadequate to accomplish these applications. A novel framework for an Intelligent and Interactive Healthcare System combining cloud computing techniques and concentrating on Speech Recognition was proposed in^8^. Here, the main emphasized on predicting physical or psychological discomfort via a Hidden Markov model with improved accuracy. However, with high user mobility precision, one of the important factors in resource-constrained sensors has said to be compromised extensively. Here Markov decision^9^ process was employed in acquiring optimal service migration policies extensively.

A review of machine learning techniques for healthcare IoT was investigated in^10^. Yet another systematic review on optimization techniques to focus on the scheduling aspects employing machine learning and data-driven approaches was designed in^11^. A deep dive into healthcare consisting of seamless connectivity, network scalability, and data privacy was analyzed alongside methods presented in^12^. Nevertheless, traditional cloud-based methods pose issues owing to the increase in volume and time-sensitive data. In^13^, a Regional Computing (RC) framework for the management of healthcare bid data was presented in detail.

One of the main advancements used in recent years is the Internet of Medical Things (IoMT) in the cloud, which permits users to access cloud services over the Internet in a remote fashion. A scheduling mechanism that addresses the Quality of Service parameters with minimal energy consumption is said to be the need of the hour. An overview of the integration of IoMT and cloud computing frameworks employing Extended Water Wave Optimization (EWWO) task scheduling in the IoMT for healthcare applications was presented in^14^. Employing this extended algorithm resulted in the improvement of execution time with improved resource utilization. Yet another method employing the augmented moth flame optimization technique to focus on the task offloading with minimal energy consumption was proposed in^15^.

### Problem definition

Many scheduling process is developed in the smart healthcare sector. Here, two existing methods are used in the proposed method performance evaluation in smart healthcare networks. At first, Deep Reinforcement Learning based computational task offloading failed to consider the involved rate of accuracy and time during the scheduling process. Another one is the Hybrid Multi-objective Optimization technique that only handles constrained resources problems. But, it failed to consider the latency and makespan involved in the healthcare sector via mobile computing services.

To concern these issues, with the assist of separate optimization and scheduling technique, the proposed KHSAROSS method design Snow Ablation-based Resource Optimization and Stride Scheduling model focused. Followed by, Kademlia Hash Function is applied to produce hash value for each patient collected data to focus the latency and makespan of overall performance.

A key objective is to construct a mobile phone based remote healthcare monitoring to identify the resource optimized virtual machine and balance the overloaded virtual machine to the less loaded virtual machine. In order to achieve this, a systematic analysis of KHSAROSS mobile computing services is performed in the CC environment. Moreover, we analyze the requirements and consequences of utilizing quality of service in terms of scheduling accuracy, scheduling time, makespan, throughput, and latency in the design of the proposed KHSAROSS method for mobile computing services in healthcare.

### Contributions of the work

As presented in the above section, there are several works in the literature in regards to mobile computing healthcare services in the CC environment. This research proposes the following novel contributions.

- To overcome resource limitations and make scheduling efficient, a method using mobile devices that integrates patient data that integrated with the scheduling process called KHSAROSS is developed based on three major processes, namely data collection task, optimization, and scheduling.
- First, the Kademlia Hash Function-based data collection model is proposed in the KHSAROSS method for generating node ID, initialization, and fine-tuning of the routing table, network joining, and finally storing the data in a cloud server for the corresponding cloud user’s requested tasks from the MMASH Datasets. The generation of node ID for each participant employing Division Hashing function and network joining process via distributed hash table is performed in a greedy fashion to minimize makespan and latency.
- To improve scheduling accuracy, scheduling time along with throughput improvement, the Snow Ablation-based Resource Optimization and Stride Scheduling model. Here, optimization and scheduling is performed separately via a fitness function (minimization of load and maximization of resource utilization). The proposed P Snow Ablation-based Resource Optimization and Stride Scheduling model using the QoS factors aids in improving the overall throughput in an efficient manner.
- Finally, a comprehensive experimental assessment is carried out with different types of performance parameters to analyze and validate the performance improvement of the proposed KHSAROSS method over conventional methods. The proposed KHSAROSS method is implemented in Python high-level general-purpose programming language, using hardware requirements such as 8 GB RAM, a 2 TB hard drive, and a CORE i7 CPU.

### Organization of the work

The rest of this work is structured as follows. Section "Related works" discusses different mobile computing services in the healthcare domain with an overview of related work and narrates their indispensable features. Section "Methodology" describes the proposed KHSAROSS method and presents strategies for algorithms. Section "Results and discussion" provides a detailed experimental setup, followed by simulation results and comparative comparisons with the state-of-the-art methods. Finally, Sect. "Conclusion" provides the conclusions of this research.

## Related works

Healthcare systems are swiftly progressing owing to the application of sophisticated techniques like, ML, IoT, a portion of AI concentrated on enabling machines to learn from data and enhance their performance to enable faster processing. These evolutions are said to be fine tuning healthcare services being managed and delivered by patients and providers alike.

The role and influence of new techniques being applied to healthcare systems were investigated in^16^. Constrained probabilities of mobile devices, in terms of processing, time, energy consumption, and storage, cause issues concerning energy optimization and time management during the processing of tasks on mobile phones. These issues increase the need for offloading to mobile devices. A novel task scheduling method to minimize energy consumption and time using an energy-efficient dynamic decision-based method was proposed in^17^. With this type of design, not only was the task scheduling time but also the energy consumption was reduced significantly. Yet another smart and secure IoT framework mobile healthcare sector was investigated in^18^.

The consolidation of healthcare and technological innovation has seen mushroom improvement inspired by the ceaseless pursuit for effectiveness, accuracy and treatment personally. As this development evolves, the drastic issue of controlling enormous amount of data and computational necessitates becomes more acute.

The method^19^ aspires to focus on the real-time healthcare demands employing dynamic offloading techniques using Digital Twins (DT) and social health determinants to improve healthcare interferences. Yet another method employing AI for Mobile Cloud Computing (MCC) in healthcare was presented in^20^. As the evolution and application of mobile cloud computing techniques in healthcare, novel optimization mechanisms have been analyzed and validated to assist mobile cloud healthcare services in being deployed in a more efficient fashion.

In^21^, a survey of mechanisms demonstrated in various healthcare applications was investigated in detail. Yet another qualitative examination was performed in^22^ for a community health worker to analyze contextual factors involved in India. Mobile health interferences could have advantageous influences on health care delivery processes. The IoT is indispensable in innovative applications, to name a few, being smart homes, healthcare, smart cities, and so on. IoT applications are specifically advantageous for imparting healthcare owing to the reason that they enable real-time remote patient monitoring to boost quality of life.

A systematic review of the efficiency of mobile health technologies for the corresponding service delivery process was investigated in^23^. Yet another investigation of improving the quality of life concerning the healthcare monitoring system was designed in^24^. A case analysis of implementing survival among Indians associated with mobile health technology was investigated in^25^. A method to focus on the important health metrics on real-time cloud computing employing a web-based interface was designed in^26^.

A review of the CC environment in healthcare systems and its influence on bioinformatics research was investigated in^27^. Yet another mobile computing services framework to focus on the accuracy and precision aspects involved in an enhanced healthcare system was designed in^28^. A holistic review of data storage mechanisms for cloud computing in healthcare was presented in^29^. A decentralized architecture was designed in^30^ for effective content delivery in use crowd-sourced video streaming using Kademlia information-sharing and exchange method. But, it failed to evaluate the real-world events’ performance effectiveness when using a larger number of nodes. Also, decentralized multi-view stream editing implementation was challenging. To address this, Kadabra, a decentralized protocol was designed in^31^ to learn the routing tables in Kademlia for increasing the speed of lookups. Here, an integration of a fully decentralized and non-cooperative algorithm was termed as Kadabra that adapts to a heterogeneity network.

Except for the above works, most other research only failed to focus on either minimizing the makespan or minimizing the latency. Different from the research aforementioned, optimal KHSAROSS aims at minimizing the makespan latency along with the overall throughput improvement. The elaborate description of the KHSAROSS method is provided in the following sections.

## Methodology

**Fig. 1:**
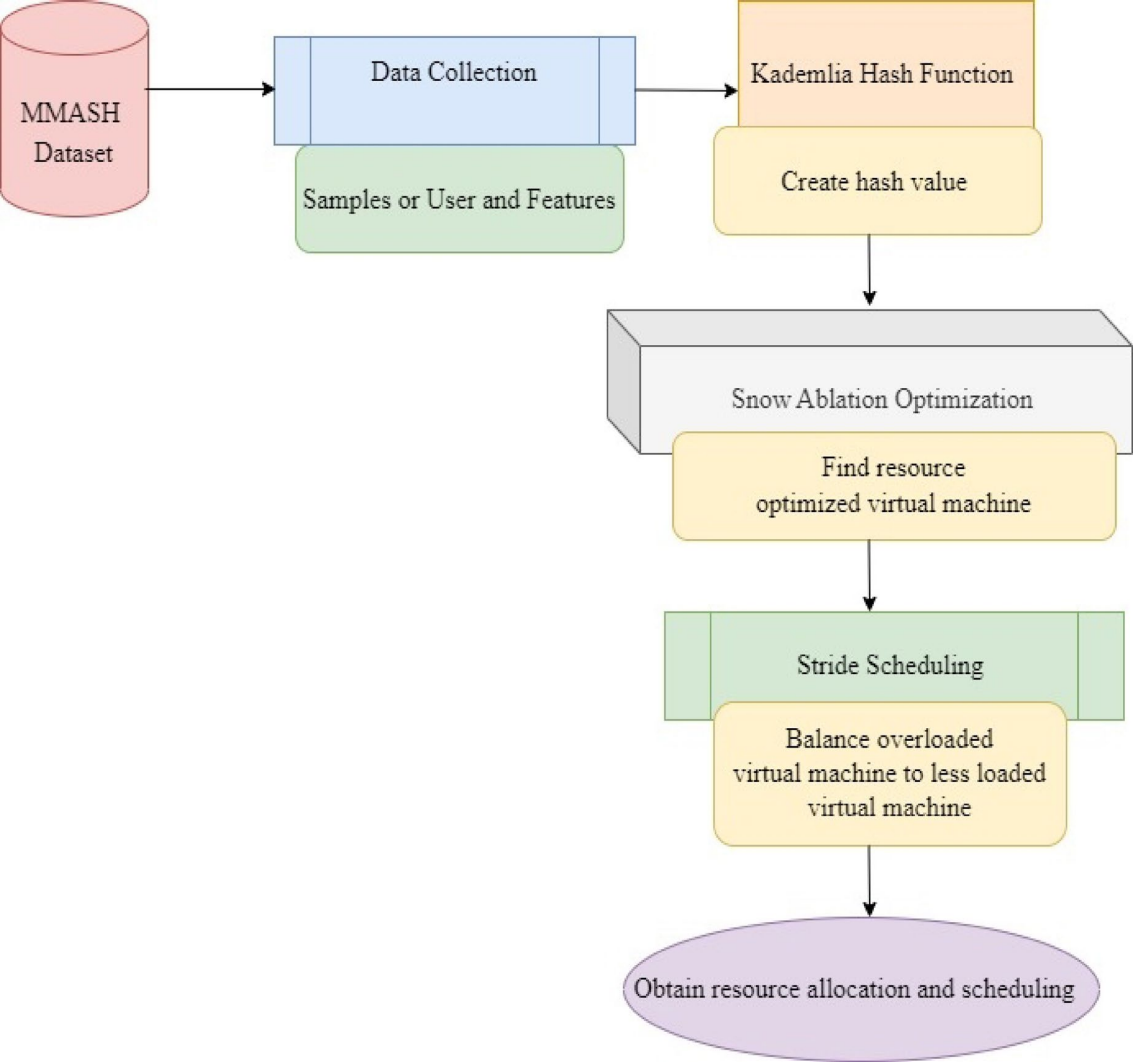
Structure of KHSAROSS for mobile computing services in healthcare sector.

As far as healthcare is concerned, mobile computing is reconstructing the process of medical services, allowing real-time access to patient data, therefore strengthening communication between healthcare providers and smoothing the path of telemedicine. However, strategic management of technology resources like data, CPU, network bandwidth, and memory to ensure effective patient care delivery still remains in infancy. In this section, a method called KHSAROSS in mobile computing is designed to focus on the resource optimization strategy therefore resulting in the improvement of efficient resource allocation and scheduling in mobile computing. Figure 1 shows the block diagram of the KHSAROSS method.

Figure 1 represents the Structure of the KHSAROSS method for mobile computing services in the healthcare sector. The KHSAROSS method is split into three processes. Initially, the patient data collection task is performed employing the MMASH dataset. The proposed method input parameters such as CPU, network bandwidth, and memory, are virtual machines or healthcare service providers. The objective function value of the proposed method is minimum load and maximum resource utilization. The Kademlia Hash Function-based data collection task is performed to protect and keep patient data privacy with the assistance of node ID generation, routing table initialization, network joining, and routing data storage in a cloud server. Here, the Kademlia Hash Function is applied to generate a hash value for each patient collected data and stored on cloud servers to scale resource allocation based on demand. Second, Snow Ablation Optimization is carried out with the intent of identifying the resource optimized virtual machine for performing scheduling of stored patient data. Third and finally, Stride Scheduling is used to balance overloaded virtual machines with less loaded virtual machines with the objective of making certain efficient resource allocation and scheduling in mobile computing.

### Kademlia hash function-based data collection

Multilevel Monitoring of Activity and Sleep in Healthy People (MMASH) dataset from Physionet (https://physionet.org/content/mmash/1.0.0/) imparts 24 h of continuous sleep quality, beat-to-beat heart data, physical activity, triaxial accelerometer data, and psychological features, to name a few, including anxiety status, emotions, and stress events for 22 healthy participants. In addition, saliva bio-markers like cortisol and melatonin, along with an activity log are also included in the dataset, hence correlating between physical activity, sleep quality, and psychological characteristics. The data were collected and provided by BioBeats in association with researchers from the University of Pisa, who generate IoT wearable devices striving to detect people’s psycho physiological stress. At the outset of the data recording process, certain anthropomorphic features like age, height, and weight of the participants were recorded. In addition, a set of inceptive questionnaires that dispense information about participants’ psychological status was also recorded. During the test, participants wore two devices for 24 h, namely, a heart rate monitor to record heartbeats and beat-to-beat interval and an actigraph to record actigraphy information like accelerometer data, sleep quality, and physical activity. Both Positive and Negative Affect Schedules were also recorded at different times of the day. Twice a day, the participants acquired saliva samples at home in pertinent vials. Saliva samples were used to extract RNA and to assess specific hormones. In the proposed KHSAROSS method, the number of patient data is collected using the MMASH dataset. The size of the MMASH dataset is 23.5 MB. Patient data is being collected from the MMASH dataset in Table 1.

**Table 1:** MMASH dataset.

S. No	Files	Description along with features
1	User_info[anthropocentric characteristic]	1. **Gender -** M and F refer to Male and Female
		2. **Height-** It denoted in centimetre (cm)
		3. **Weight-** It measured expressed in kilograms (kg)
		4. **Age –**It expressed in years
2	Sleep [participant sleep duration and sleep quality]	1. **In bed date**- 1 and 2 define to the first and second day of data recording.
		2. **In bed time**-time of day (hours, minutes) when the user go to the bed.
		3. **Out bed date**-1 and 2 define to the first and second day of data recording
		4. **Out bed time**-time of day (hours, minutes) when the user go out of the bed.
		5. **Onset date**- 1 and 2 define to the first and second day of data recording
		6. **Onset time**-time of day (hours, minutes) when the user falls asleep
		7. **Latency efficiency**-ratio of sleep time on whole sleep in bed
		8. **Total minutes in bed**-Minutes exhausted in the bed per night
		9. **Total sleep time**-Duration of the sleep per night expressed in minutes.
		10. **Wake after sleep onset**-time spent awake after falling asleep the first time.
		11. **Awakenings during night**
		12. **Average awakening length**-time in seconds spent awakening through the night.
		13. **Movement index**-number of minutes without movement denoted as a percentage of the movement phase
		14. **Fragmentation index**-number of minutes with movement represented as a percentage of the immobile phase
		15. **Sleep fragmentation index**-proportion among the Movement and Fragmentation indices.
3	RR [beat-to-beat interval]	1. **ibi_s** - time in seconds among two consecutive beats
		2. **day** − 1 and 2 define to the first and second day of data recording
		3. **time** - day time when the heartbeat happened
4	Questionnaire [scores for questionnaire]	1. **MEQ** – Morningness - Eveningness Questionnaire value
		2. **STAI1**-State Anxiety value achieved from State-Trait Anxiety Inventory
		3. **STAI2**-Trait Anxiety value attained from the State-Trait Anxiety Inventory
		4. **PSQI**-Pittsburgh Sleep Quality Questionnaire Index
		5. **BIS/BAS**-Behavioural avoidance/inhibition index
		6. **Daily stress**- Daily Stress Inventory value (DSI)
		7. **PANAS-**Positive and Negative Affect Schedule
5	Activity [activity categories throughout day]	1. Sleeping
		2. Laying down
		3. Sitting
		4. Light movement
		5. Medium
		6. Heavy
		7. Eating
		8. Small screen usage
		9. Large screen usage
		10. Drink consumption
		11. Smoking
		12. Alcohol consumption
6	Actigraph [accelerometer and inclinometer data]	1. **Axis 1**-Raw Acceleration data of the X-axis denoted in Newton-meter.
		2. **Axis 2**-Raw Acceleration data of the Y-axis represented in Newton-meter.
		3. **Axis 3**-Raw Acceleration data of the Z-axis indicated in Newton-meter.
		4. **Steps**-Number of steps per second.
		5. **HR**-Beats per minutes (bpm).
		6. **Inclinometer off-**Values equal to 1 define to no activation of the inclinometer. The values are reported per second.
		7. **Inclinometer sitting**-values equal to 1 denoted to the standing position of the user and 0 indicated to other user positions. Values are reported per second.
		8. **Inclinometer lying**-values equal to 1 represented to the sitting position of the user and 0 indicated to other user positions. Values are reported per second.
		9. **Vector magnitude**-Vector movement derived from raw acceleration data denoted in Newton-meter.
		10. **Day**- 1 and 2 define to the first and second day of data recording
		11. **Time**- day time when the heartbeat happened
7	Saliva	Clock genes, hormone concentrations [two samples]/ person are included

**Fig. 2:**
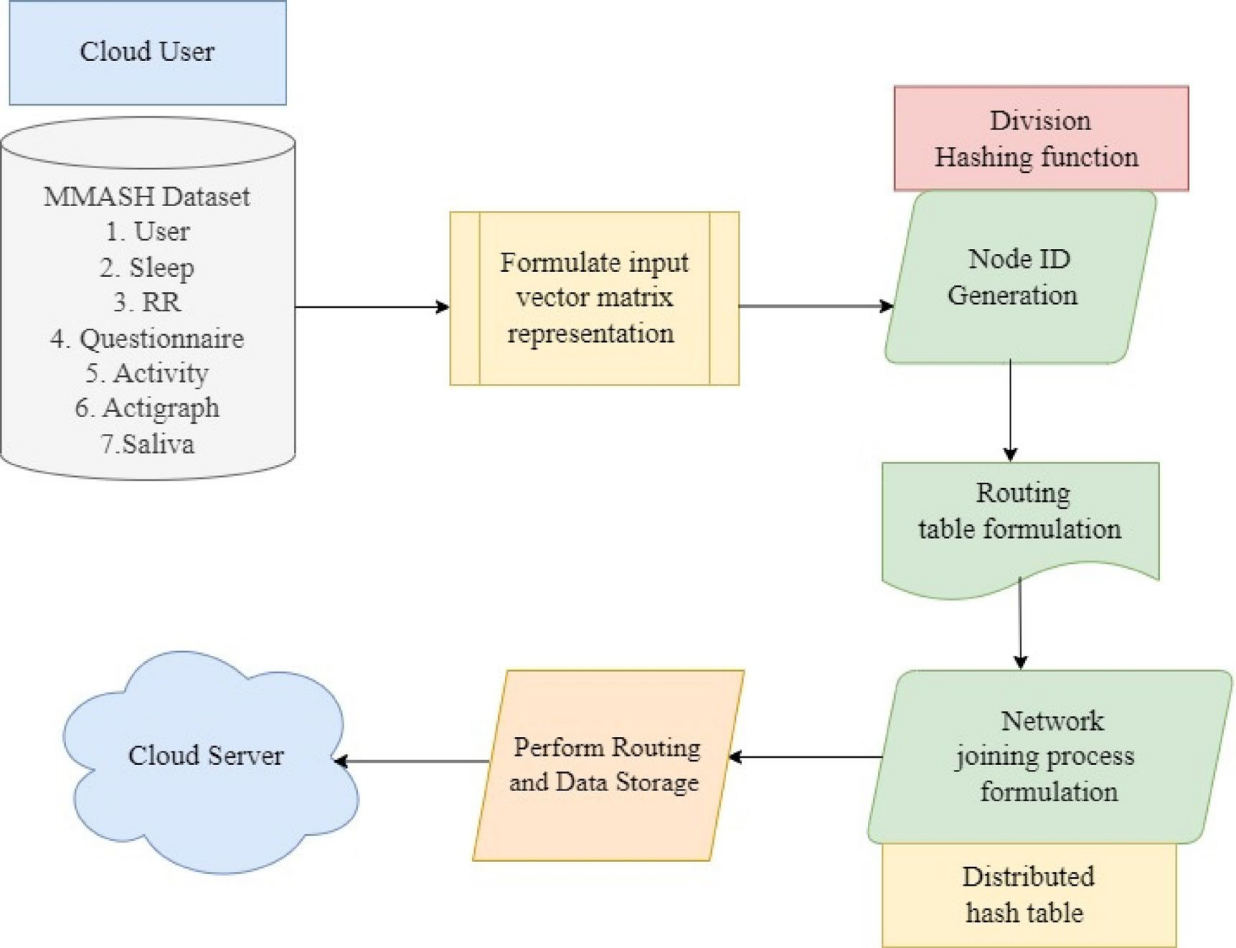
Structure of Kademlia Hash Function-based data storage.

Collected data is used to perform two processes, namely data normalization and feature selection. In the data obtaining step, sensors are used to gather the patient’s physiological conditions like temperature, heart rate, and EEG data to carry out mobile phone-based remote healthcare monitoring. After data collection, the Canberra Match data normalization based Pre-processing technique is applied to process the raw dataset into a structured format. It helps to increase the quality of the data, provides more accurate analytics and decision-making for achieving improved patient care, and more effective healthcare functions. Once data normalization is completed, relevant feature selection is performed to select only the most pertinent features from the input data using the Elasticnet Regressive Attribute Selection technique. Thus, it achieves higher scheduling accuracy with minimal time consumption. With the above set of selected features, the Kademlia Hash Function is applied to each patient’s collected data to generate a corresponding hash value. Figure 2 shows the structure of the Kademlia Hash Function-based data collection model.

Figure 2 illustrates that the Kademlia Hash Function-based data storage on a cloud server involves around four steps, namely, generation of node ID, routing table initialization, network joining, and routing data storage in the cloud server. Let us consider the MMASH dataset ‘$\:DS$’ with ‘$\:N$’ samples or users corresponding to seven different files (i.e., user information, sleep, RR, questionnaire, activity, actigraph, and saliva) for each participant with an overall of ‘$\:69$’ features. The information in the dataset is mathematically formulated as given below.

1 $$\:DS\to\:\left\{user,\:sleep,\:RR,\:questionnaire,\:activity,\:actigraph,\:saliva\right\}$$

The above seven files present in the dataset are then split in the form of an input vector matrix representation as given below.

2 $$\:IM=\left[\begin{array}{cccc}{S}_{1}{F}_{1}&\:{S}_{1}{F}_{2}&\:\dots\:&\:{S}_{1}{F}_{M}\\\:{S}_{2}{F}_{1}&\:{S}_{2}{F}_{2}&\:\dots\:&\:{S}_{2}{F}_{M}\\\:\dots\:&\:\dots\:&\:\dots\:&\:\dots\:\\\:{S}_{N}{F}_{1}&\:{S}_{N}{F}_{2}&\:\dots\:&\:{S}_{N}{F}_{M}\end{array}\right]$$

From the above formulation according to Eq. (2), the input vector matrix ‘$\:IM$’ is generated with respect to ‘$\:N$’ samples ‘$\:{S}_{N}$’ and ‘$\:M$’ features ‘$\:{F}_{M}$’. Let us consider a Kademlia network of ‘$\:N$’ samples with IDS ‘$\:{S}_{1},\:{S}_{2},\:\dots\:{S}_{N}$’ each of which denotes a string length ‘$\:s$’corresponding to ‘$\:1$’ and ‘$\:0$’. Kademlia network of ‘$\:N$’ samples in the proposed method is modeled as a trie or prefix tree, wherein each lead denotes a node, whereas the labeled path between the root and a lead denotes its ID. Then, the generation of node ID for each participant employing the Division Hashing function is formulated as given below.

3 $$\:Key\to\:H\left(IM\right)=IM\left(modTS\right)\to\:Hashvalue$$

From the above formula, according to Eq. (3), the Division Hashing function ‘$\:H$’ results for the corresponding input matrix ‘$\:IM$’ are arrived at based on the modulo function ‘$\:mod$’ by selecting a divisor ‘$\:TS$’, a prime number close to the table size. Then, for a sample of input vector matrix ‘$\:IM\in\:\left\{{IM}_{1},\:{IM}_{2},\:\dots\:,{IM}_{n}\right\}$’, let ‘$\:{s}_{i}\left(IM\right)$’ denote the set of samples of input vector matrix (IDs) that share a prefix with ‘$\:IM$’ of length ‘$\:s-i$’. Nodes or the samples initiate a routing table employing ‘$\:k$’ structure as a table with every bucket containing contact information for other nodes or samples with their IP address and node ID, respectively. Then, filling the ‘$\:i-th$’ bucket is said to be modeled as inserting pointers from the leaf ‘$\:IM$’ to ‘$\:k$’ leaves arbitrarily. Hence, routing is said to be jumping among leaves along these pointers in such a manner so as to go towards the target ID to the fullest extent in a greedy fashion, as given below.

4 $$\:RT\left[IM\right]\to\:Adding{IM}_{i}\left[H\left(IM\right)\right]to{IM}_{k}\left[H\left(IM\right)\right]$$

With the above routing table formulated according to Eq. (4), when a node or sample becomes a member, contact with other samples is said to be initiated, so that network joining is said to be ensured robustly. Let ‘$\:{J}_{xy}$’ represent the number of jumps required to traverse from leaf ‘$\:x$’ to target ‘$\:y$’. Then, the network joining process via a distributed hash table is mathematically represented as given below.

5 $$\:\underset{{{IM}}_{1},\:{{IM}}_{2},\:\dots\:,{{IM}}_{n}}{\text{sup}}\underset{{IM}\in\:\left\{{{IM}}_{1},\:{{IM}}_{2},\:\dots\:,{{IM}}_{n}\right\}}{\text{sup}}\underset{y\in\:{\left\{\text{0,1}\right\}}^{s}}{\text{sup}}E\left[{J}_{xy}\right]\le\:\left(1+o\left(1\right)\right)\frac{\text{log}n}{{H}_{k}}$$

From the above Eq. (5) ‘$\:{H}_{k}$’ denotes the harmonic number where only ‘$\:O\left(\text{log}\left(n\right)\right)$’samples are contacted in searching for a target node to be stored in the routing table. Finally, the routing and data storage in the cloud server are performed as given below.

6 $$\:{CS\to\:J}_{xy}\left[{IM}_{i}\right]=\frac{\text{log}n}{{C}_{k}}\left[{IM}_{i}\right]$$

From the above Eq. (6), the routing and data storage information for each sample or cloud user ‘$\:{J}_{xy}\left[{IM}_{i}\right]$’ via input matrix is stored in the cloud server ‘$\:CS$’ for further processing. The pseudo code representation of Kademlia Hash Function-based data collection is as given below.

**Table:** 

Input: Dataset ‘$\:DS$’, Samples or Cloud Users ‘$\:S=\left\{{S}_{1},\:{S}_{2},\:\dots\:,{S}_{N}\right\}$’, Features ‘$\:F=\left\{{F}_{1},\:{F}_{2},\:\dots\:,{F}_{M}\right\}$’
**Output**: makespan and latency-efficient data collection
Step 1: **Initialize** ‘$\:N=22$’, ‘$\:M=69$’, table size ‘$\:TS$’ Step 2: **Begin** Step 3: **For** each ‘$\:DS$’ with ‘$\:S$’ and ‘$\:F$’ Step 4: Formulate input vector matrix representation according to (1) and (2) **//Generation of Node ID** Step 5: Generate Node ID using Division Hashing function according to (3) **//Initialization of routing table** Step 6: Formulate the routing table according to (4) **//Network joining** Step 7: Formulate the network joining process according to (5) **//Routing and data storage in cloud server** Step 8: Perform routing and data storage in the cloud server according to (6) Step 9: **End for** Step 10: **End**

**Algorithm 1** Kademlia Hash Function-based data collection.

As given in the above algorithm, the data collection processing using the Kademlia Hash Function is split into four steps. Initially, with the corresponding input vector matrix representation for respective samples or cloud users, by employing the Division Hashing function, undergoes a process of generation of node ID, therefore, with fast lookup time ensures minimum makespan time. Next, according to the results of the Division Hashing function, the results routing table is generated. Followed by which network joining process for each sample or cloud users is generated via a distributed hash table. Finally, the data stored in the cloud server not only serves as the identification process but also employs the node ID in locating the corresponding information of each cloud user. This in turn, aids in minimizing the overall latency involved extensively.

### Snow ablation-based resource optimization and Stride scheduling model

**Fig. 3:**
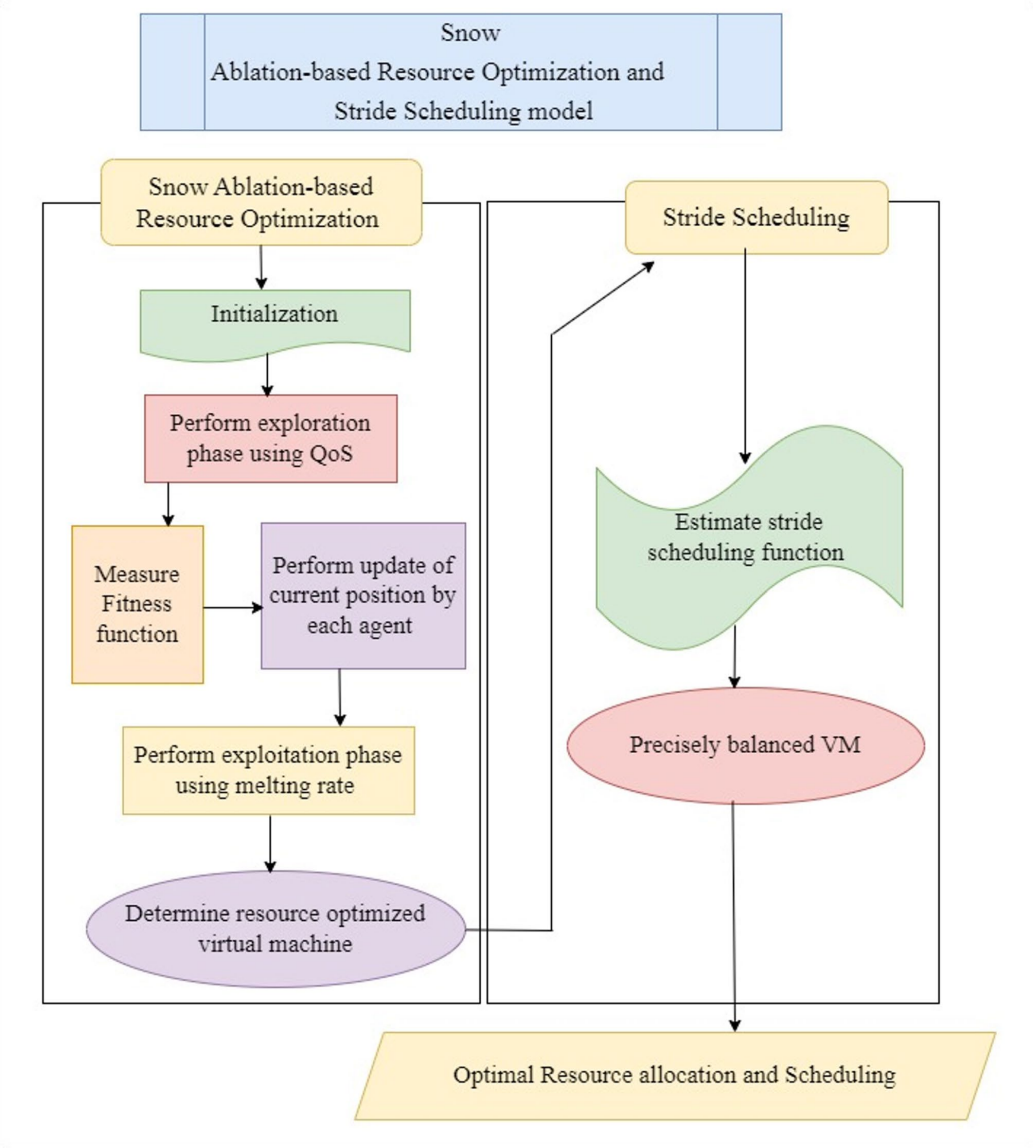
Structure of Snow Ablation-based Resource Optimization and Stride Scheduling model.

With the data collected and stored in a cloud server, the next step in the proposed method is to design a resource optimization model. In this section Snow Ablation-based Resource Optimization and Stride Scheduling model is designed. Snow Ablation Optimization is a meta-heuristic process inspired by two processes that turn snow into liquid water via melting and liquid water into steam via evaporation, permitting a balance between exploration (searching a wide range of solutions) and exploitation (concentrating on fine-tuning optimal solutions). The Snow Ablation Optimization is carried out to identify resource optimized virtual machines for performing scheduling of stored patient data. Figure 3 shows the structure of the Snow Ablation-based Resource Optimization and Stride Scheduling model.

Figure 3 illustrates the process of Snow Ablation-based Resource Optimization and Stride Scheduling model is split into two parts. In the first part, optimization is performed using Snow Ablation-based Resource Optimization, and scheduling is done employing the Stride Scheduling function. The Snow Ablation-based Resource Optimization and Stride Scheduling model is designed to increase scheduling accuracy with the help of optimization and scheduling techniques. Stride Scheduling is used in the proposed method with the purpose of balancing overloaded virtual machines with less loaded virtual machines, which makes certain efficient resource allocation and scheduling in mobile computing. This scheduling process results in precise control over proportional resource allocation between competing processes. For example, a different disease dataset is given as input to Stride Scheduling in the healthcare system. Here, the stride scheduling function estimation and precise VM balancing steps are performed. The Stride Scheduling function is estimated by the difference between the number of loads of process by a virtual machine to the large number (virtual machine). This stride scheduling process is used with more samples receiving a larger share of the resource over time, therefore achieving better throughput for that process.

#### Snow Ablation-based resource optimization model

Snow Ablation-based Resource Optimization model is constructed with three components to identify the optimal solution, i.e., the initialization phase, the exploration phase, and the exploitation phase. The iterative process of the Snow Ablation-based Resource Optimization model initiates with arbitrarily randomly generated populations. The entire arbitrarily randomly generated populations are denoted by a matrix with an organization of ‘$\:R$’ rows and ‘$\:C$’ columns as provided in Eq. (7). Here ‘$\:R$’ represents to total number of agents (i.e., virtual machines or healthcare service providers) and ‘$\:C$’ represents the dimension of solution space, with upper and lower boundaries of search domain being ‘$\:UB$’ and ‘$\:LB$’ respectively.

7 $$\:P=LB+rand*\left(UB-LB\right)=\left[\begin{array}{cccc}{p}_{11}&\:{p}_{12}&\:..&\:{p}_{1C}\\\:{p}_{21}&\:{p}_{22}&\:..&\:{p}_{2C}\\\:..&\:..&\:..&\:..\\\:{p}_{R1}&\:{p}_{R2}&\:..&\:{p}_{RC}\end{array}\right],\:where\:p\in\:P\:[i.e.data\:stored\:in\:CS]$$

Then the ‘$\:i-th$’ search agent position (i.e., samples or cloud users) is then described as ‘$\:{P}_{i}=\left({p}_{i1},\:{p}_{i2},\:\dots\:,{p}_{iC}\right)$’. Distinct candidate solutions equivalent to discrete function values, and these function values are mathematically formulated as given below.

8 $$\:F=\left[F\left({P}_{1}\right),F\left({P}_{2}\right),\dots\:,F\left({P}_{R}\right)\right]$$

In Snow Ablation Optimization, a meta-heuristic, the candidate solution quality is measured by the function value of the problem (CPU, network bandwidth, and memory). Therefore, the agent (i.e., virtual machines or healthcare service providers) analogous to the best objective function value (i.e., minimum load and maximum resource utilization) is considered the best agent. In this paper, it is assumed that the minimization (i.e., load) and maximization (i.e., resource utilization) problem is solved, and hence, the agent (i.e., virtual machines or healthcare service providers) with the minimum and maximum value of ‘$\:F$’is the best agent. Then, the load of a VM on a physical node is resources of CPU, memory, network to consuming and how that usage impacts the overall performance of the physical server. It is mathematically formulated as,

9 $$\:{L}_{{VM}_{j}}=\frac{1}{1-{CPU}_{{UR}_{j,i}}}*\frac{1}{1-{Net}_{{UR}_{j,i}}}*\frac{1}{1-{MEM}_{{UR}_{j,i}}}$$

From the above Eq. (9), ‘$\:{L}_{{VM}_{j}}$’ denotes the load of ‘$\:j-th$’ virtual machine ‘$\:{VM}_{j}$’ corresponding to ‘$\:i-th$’ physical node ‘$\:{S}_{i}$’ (i.e., cloud user) and ‘$\:{CPU}_{{UR}_{j,i}}$’, ‘$\:{Net}_{{UR}_{j,i}}$’, ‘$\:{MEM}_{{UR}_{j,i}}$’ representing the utilization rate of its CPU, network bandwidth, and memory, respectively. After that, the resource utilization is measured to utilize this rate, it’s essential to calculate it accurately, track it running time, and then use the insights to optimize resource allocation and enhance the overall efficiency and profitability. It is formulated as,

10 $$\:RUR=\sum\limits_{i=1}^{N}\sum\limits_{j=1}^{J}\frac{{UR}_{j,i}}{{f}_{i}}$$

Also, according to Eq. (10), ‘$\:RUR$’ denotes the resource utilization rate or the entire population in the sample space, and also ‘$\:f$’ represents the flag with ‘$\:{f}_{i}=1$’ denoting certain cloud user requested tasks are running on virtual machine ‘$\:VM$’ on ‘$\:i-th$’ physical node ‘$\:{S}_{i}$’ and on the contrary the flag with ‘$\:{f}_{i}=0$’ denoting certain cloud user requested tasks not running on virtual machine ‘$\:VM$’ on ‘$\:i-th$’ physical node ‘$\:{S}_{i}$’. Finally, the ‘$\:RUR$’ is improve the efficiency, lesser costs. Hence, the QoS has to meet the following conditions as given below.

11 $$\:F=\text{Min}\left({L}_{{VM}_{j}}\right)and\:Max\left(RUR\right)$$

From the above equation results Eq. (11) on the basis of minimal load and maximal resource utilization rate, the optimal virtual machine (i.e., healthcare service providers) is selected for performing scheduling of stored patient data. Eventually, when the load or the resource utilization rate is violated, reallocation or right VMs are performed via the exploration and exploitation phase.

Second, in the exploration phase, the Snow Ablation-based Resource Optimization model simulates the transition of snow and liquid water to steam with the intent of arriving at achieve an exploration of the problem space. Then, each agent (i.e., virtual machines or healthcare service providers) updates its current position as given below.

12 $$\:{P}_{i}^{t+1}={P}_{elite}^{t}+{BM}_{i}*\left({Cons}_{1}*\left({P}_{best}^{t}-{P}_{i}^{t}\right)+\left(1-{Cons}_{1}\right)*\left({P}_{mean}^{t}-{P}_{i}^{t}\right)\right)$$

From the above Eq. (12) ‘$\:{P}_{i}^{t+1}$’ denotes the position of the ‘$\:i-th$’ agent or the virtual machine in ‘$\:t+1$’ iteration, with ‘$\:{BM}_{i}$’ denoting the Brownian random numbers, ‘$\:{Cons}_{1}$’ denoting the arbitrarily generated value, ‘$\:{P}_{best}^{t}$’ representing the best solution with minimum fitness obtained so far for the current iteration ‘$\:t$’ respectively. Moreover, ‘$\:{P}_{mean}^{t}$’ and ‘$\:{P}_{elite}^{t}$’ denotes the current mean location and agents or virtual machine selected from the elite pool, mathematically formulated as given below.

13 $$\:{P}_{mean}^{t}=\frac{\sum\nolimits_{i=1}^{R}{P}_{i}^{t}}{R}$$

14 $$\:{P}_{elite}^{t}=\left[{P}_{best}^{t},{P}_{second}^{t},{P}_{third}^{t},..\right]$$

From the above Eqs. (13) and (14), ‘$\:{P}_{second}^{t}$’ and ‘$\:{P}_{third}^{t}$’ represents the second best agent or virtual machine and the third best agent or virtual machine.

Third in the exploitation stage, when the snow melts and becomes liquid water, search agents (i.e., virtual machines or healthcare service providers) are prompted to employ high-quality solutions adjacent to the current best solution. The exploitation stage of the Snow Ablation-based Resource Optimization model motivates searching agents to concentrate on exploitation encompassing the current optimal agent.

15 $$\:{MR}^{t}=\left(0.35+0.25*\frac{{e}^{\frac{t}{{t}_{max}}}-1}{e-1}\right)*{e}^{\frac{-t}{{t}_{max}}}$$

From the above Eq. (15), ‘$\:{MR}^{t}$’ represents the melting rate at ‘$\:t-th$’ iteration and accordingly the position update to identify resource optimized virtual machine for performing scheduling of stored patient data is mathematically formulated as,

16 $$\:{P}_{i}^{t}={MR}^{t}*{P}_{best}^{t}+{BM}_{i}*\left({Rand}_{2}*\left({P}_{best}^{t}-{P}_{i}^{t}\right)+\left(1-{Rand}_{2}\right)*\left({P}_{mean}^{t}-{P}_{i}^{t}\right)\right)$$

From above Eq. (16), the ‘$\:{P}_{i}^{t}$’ is position update to ‘$\:{P}_{best}^{t}$’ denotes the best solution, ‘$\:{P}_{mean}^{t}$’ denotes the current mean location and agents or virtual machine selected from stored patient data. It utilized to schedule and optimize the storage and retrieval of patient data in a resource-efficient manner. By using Kademlia hash function, patient data can be ensuring high availability and fault tolerance. The hash function within Kademlia data to specific nodes, enabling efficient data location and retrieval based on the hashed identifiers. The proposed KHSAROSS method carried out normalization after data collection. It increases the accuracy of the designed process. Then, the hash value for each patient collected data using Kademlia hash function to optimize resources and then virtual machine to achieve better performance of patient data scheduling process. It improves the resource utilization by distributing the storage and processing load and can also enhance data security and privacy through decentralized storage.

#### Stride scheduling

Finally, Stride Scheduling is utilized in the proposed method with the intent of balancing overloaded virtual machines to less loaded virtual machines. This in turn, makes certain efficient resource allocation and scheduling in mobile computing. The Stride Scheduling is mathematically formulated as given below.

17 $$\:SSf=\frac{a\:large\:Equation\:Number\left[virtual\:machine\right]}{the\:Equation\:Number\:of\:loads\:of\:process\:by\:virtual\:machine\:}=\frac{{VM}_{LN}}{{VM}_{L}}$$

From the above Eq. (16), the Stride Scheduling function ‘$\:SSf$’ results are arrived at based on the large number of virtual machines in the simulation setup ‘$\:{VM}_{LN}$’ and the number of loads by virtual machine ‘$\:{VM}_{L}$’. In this manner, Stride Scheduling function results aids in balancing overloaded virtual machines to less loaded virtual machines precisely and accurately. The pseudo-code representation of Snow Ablation-based Resource Optimization and Stride Scheduling is given below.

**Table:** 

Input: Dataset ‘$\:DS$’, Samples or Cloud Users ‘$\:S=\left\{{S}_{1},\:{S}_{2},\:\dots\:,{S}_{N}\right\}$’, Features ‘$\:F=\left\{{F}_{1},\:{F}_{2},\:\dots\:,{F}_{M}\right\}$’
**Output**: computationally-efficient and throughput improved scheduling
Step 1: **Initialize** ‘$\:N=22$’, ‘$\:M=69$’ Step 2: **Initialize** ‘$\:rand\:between\:0\:and\:1$’, ‘$\:{Cons}_{1}\:between\:0\:and\:1$’, ‘$\:{Rand}_{2}\:between-1\:and\:1$’ Step 3: **Begin** Step 4: **For**‘$\:DS$’ with ‘$\:S$’ and ‘$\:F$’ **//Fetch details of routing and data storage in cloud server** **//Initialization phase** Step 5: Perform the initialization process according to (7) and (8) **//Exploration phase** Step 6: Formulate fitness function according to (9) and (10) Step 7: Formulate QoS$\:\:{\prime\:}F=\text{Min}\left({L}_{{VM}_{j}}\right)and\:Max\left(RUR\right){\prime\:}$ Step 8: Perform an update of the current position by each agent according to (12), (13), and (14) **//Exploitation phase** Step 9: Formulate exploitation encompassing the current optimal agent using the melting rate according to (15) and (16) Step 10: Return resource optimized virtual machine Step 11: **End for** Step 12: **For** each ‘$\:DS$’ with ‘$\:S$’ and ‘$\:F$’ with resource optimized virtual machine **//Scheduling** Step 13: Evaluate $\:SSf$according to (17) Step 14: Return balanced scheduled VM results Step 15: **End for** Step 16: **End**

**Algorithm 2** Snow Ablation-based Resource Optimization and Stride Scheduling.

According to the above algorithm, the overall process is split into two parts, namely optimization and scheduling. Initially, the process of optimization is split into three portions, namely, initialization, exploration, and exploitation. Here, initially, details of routing and data stored in the cloud server are obtained. Following which, the initialization process is triggered according to the upper and lower boundaries of the search domain. Following which, the fitness function is generated according to the QoS factors (i.e., minimum load and maximum resource utilization rate) in the exploration stage. Based on the fitness function results position updated is made. Finally, the exploitation function is performed employing the melting rate, therefore returning resource optimized virtual machine both accurately and minimum time. On the other hand scheduling process is carried out using the Stride Scheduling function. By applying these functions result in achieve precise control over the proportional allocation of resources between competing processes, ensuring deterministic fairness. This in turn, aims to allocate resources proportionally to the cloud user requests assigned to each process, prompting a direct association between cloud user requests and the process’s throughput. In other words, a scheduling process with more cloud user requests or samples receives a larger share of the resource over time, hence achieving higher throughput for that process.

## Results and discussion

This method introduces a resource-efficient mobile computing service in healthcare sector. The study used the MMASH dataset^32^. The proposed method is executed in Python high-level general purpose programming language. The hardware requirements of the model implementation include 8 GB RAM, a 2 TB hard drive, and a CORE i7 CPU. The results were assessed with regard to scheduling accuracy, scheduling time, latency, throughput, and makespan.

### Training and testing performance

The KHSAROSS’s performance is evaluated by splitting the input MMASH dataset into a 80:20 ratio for training and testing. Table 2 lists the implementation parameters and their specifications.

**Table 2:** Parameter specifications.

S. No	Parameters	Specifications
1	Dataset name	MMASH dataset
2	Dataset link	https://physionet.org/content/mmash/1.0.0/
3	Dataset size	99.2 MB
4	Data packets	
5	Number of virtual machines	10, 20, 30, 40, 50, 60, 70, 80, 90, 100
6	Training ratio	80
7	Testing ratio	20
8	Implementation platform	Python
9	Version	3.10.11

### Performance comparison with current methods

In this section, the results obtained using the proposed KHSAROSS method were compared to those achieved with existing methods, including OO-DRL^1^ and HMHHO^2^. The outcomes metrics used for comparative evaluation include scheduling accuracy, scheduling time, makespan, latency, and throughput. The performance of these methods is arrived at by modeling and implementing them on the Python version 3.10.11 (same implementation tool used for evaluating the proposed method), for the common input MMASH dataset. Similar to implementing the proposed method, these existing methods^1^ and ^2^ will be trained and tested using the input based on Python 3.10.11. The results are determined by incrementing the sample size from 6000 to 60,000 (i.e., the actigraph of each user). For each sample size, analyze the above-stated outcome metrics that enable us to determine how the methods respond to different samples for mobile computing services.

#### Performance analysis of Makespan and latency

As far as cloud computing is concerned, makespan refers to the total time it consumes in accomplishing all the cloud users’ requested tasks within a given workload. To be more specific, makespan refers to the time taken from the start of the first cloud user’s requested task to the completion of the last cloud user’s requested task. On the other hand, latency denotes the delay encountered when sending healthcare information between the cloud user and the cloud server. To be more specific, the latency rate refers to the time it takes for a single cloud user’s requested data packet to travel from one point to another on the cloud computing network.

18 $$\:MS=\sum\limits_{i=1}^{N}{S}_{i}*Time\:\left(St-Ft\right)$$

From the above Eq. (18), Makespan ‘$\:MS$’ essentially refers to the time from when the first task starts ‘$\:St$’ to when the last task finishes ‘$\:Ft$’. The lower the makespan more efficient the method is said to be and vice versa. Makespan is measured in terms of seconds (sec).

19 $$\:Lat=\sum\limits_{i=1}^{N}{S}_{i}*Time\left[S-CS\right]$$

From the above Eq. (19), latency is ‘$\:Lat$’ refers to the delay between a cloud user’s request (samples) ‘$\:Time\:\left(S\right)$’ and the response from the cloud server ‘$\:Time\:\left(CS\right)$’. It is measured in terms of seconds (sec).

**Fig. 4:**
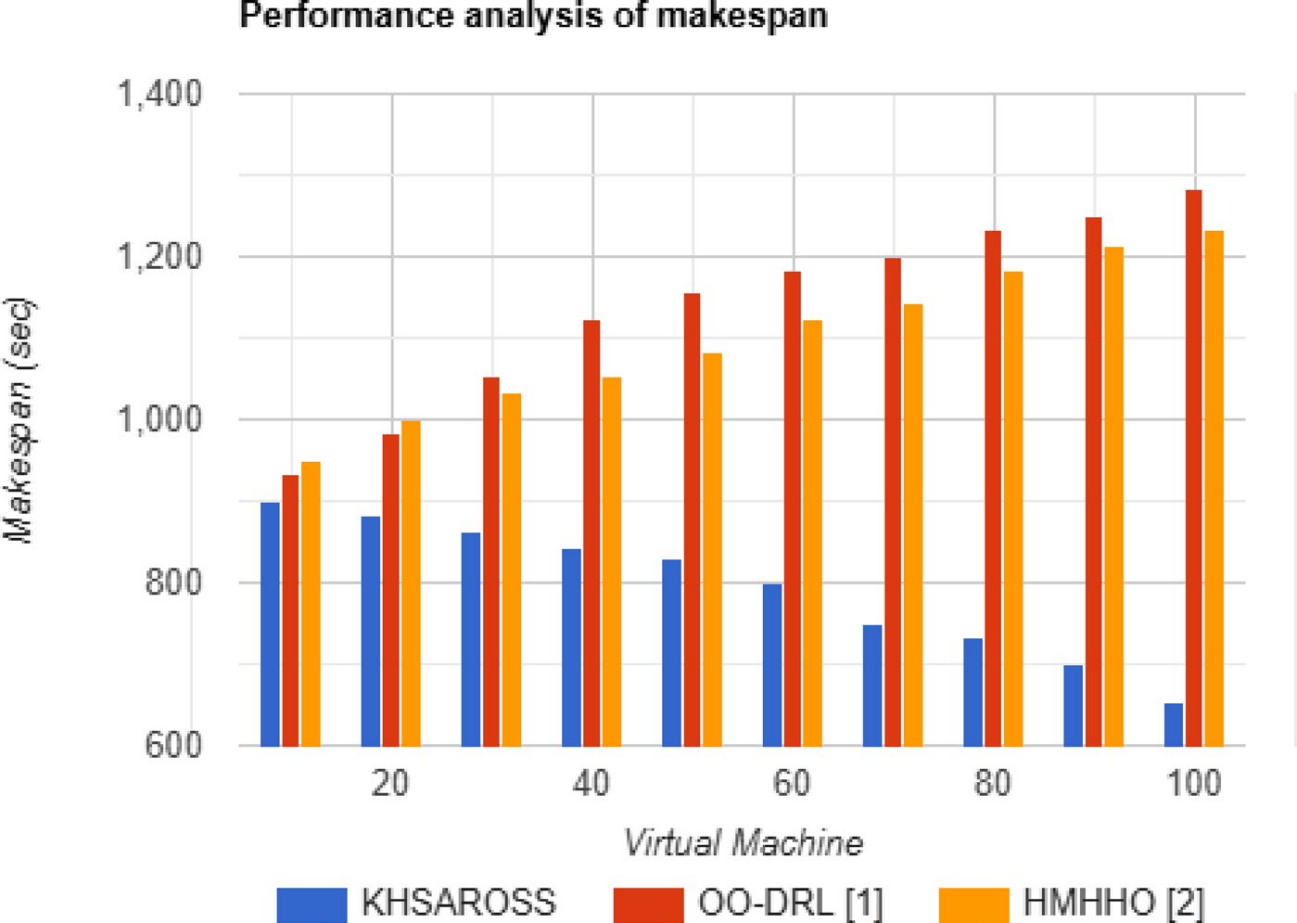
Makespan evaluations of existing methods with the proposed method.

**Fig. 5:**
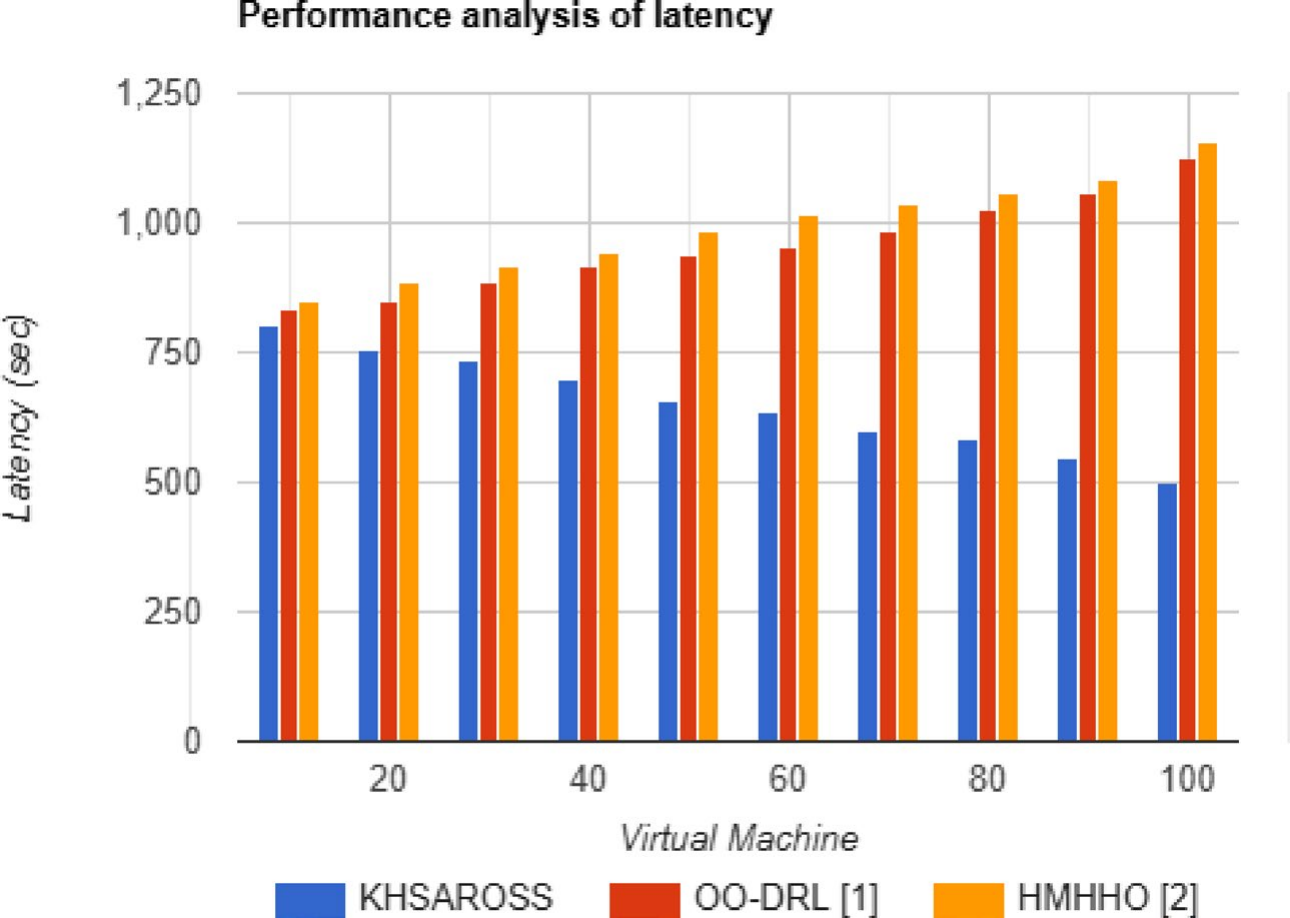
Latency evaluations of existing methods with the proposed method.

Figures 4 and 5 given above show the makespan and latency evaluation using the proposed KHSAROSS and two existing methods, OO-DRL^1^ and HMHHO^2^. The horizontal axis in the above two figurative representations denotes the virtual machines used for simulation. From both of the above graphical representations, increasing the number of virtual machines results in a decrease of makespan and latency because the higher the number of virtual machines, the greater the probability of allocating resources in an efficient manner and vice versa. Also, simulation results show betterment of makespan and latency improvements using the proposed KHSAROSS method upon comparison to^1^ and ^2^. The reason behind the improvement was owing to the application of the Kademlia Hash Function-based data collection algorithm. By applying this algorithm with the use of the Division Hashing function, the corresponding input vector matrix representations for respective samples or cloud users were obtained. This in turn, ensured fast lookup time, therefore reducing the makespan time using the proposed KHSAROSS method by 29% compared to^1^ and 27% compared to^2^. In addition, based on the Division Hashing function results routing table was generated. Finally network joining process for each sample or cloud user was generated using a distributed hash table. This in turn, aids in reducing the overall latency of the proposed KHSAROSS method by 31% compared to^1^ and 33% compared to^2^.

#### Performance analysis of scheduling accuracy

Accurate scheduling is pivotal for effective mobile computing cloud operations, making certain cloud user requested tasks are completed on time, employing resources efficiently, and delivering optimal performance to cloud users. To be more specific, scheduling accuracy in mobile computing cloud operations refers to the degree to which the scheduling algorithm precisely allocates cloud user-requested tasks to the most pertinent available resources within the CC environment. Scheduling Accuracy‘$\:SA$’ is mathematically represented as given below.

20 $$\:SA=\sum\limits_{i=1}^{N}\frac{{S}_{SA}}{{S}_{i}}*100$$

From the above Eq. (20), $\:SA$’ is measured based on the cloud user’s requested tasks or samples in the queue.$\:{S}_{i}$’ and the samples are scheduled accurately according to the requirements ‘$\:{S}_{SA}$’. Scheduling accuracy more efficient the method is said to be higher, and vice versa. It is measured in terms of percentage (%).

**Fig. 6:**
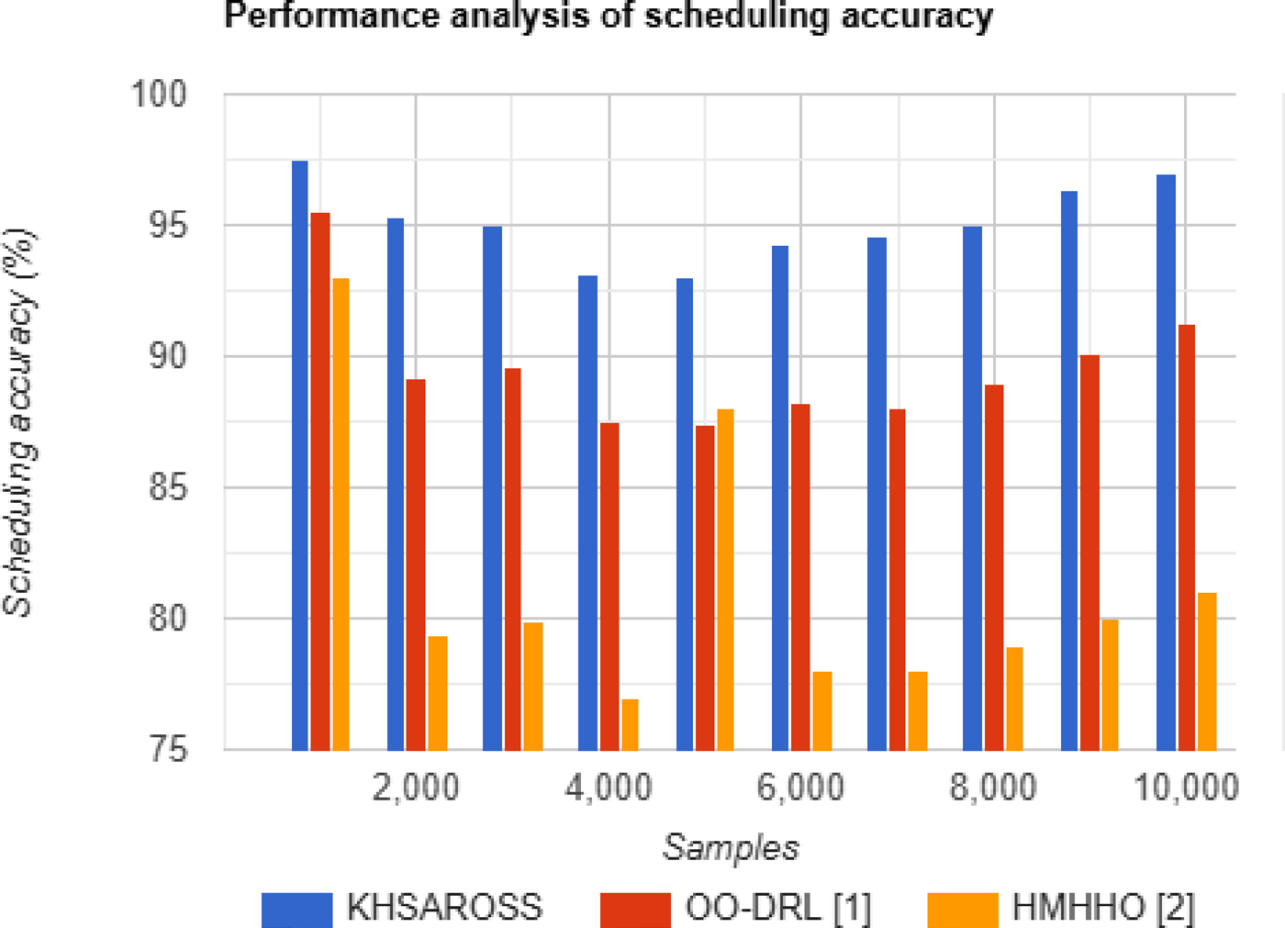
Scheduling accuracy evaluations of existing methods with the proposed method.

Figure 6, given above, illustrates the scheduling accuracy involved in the mobile computing services in healthcare. The horizontal axis in the above figure represents the samples or the cloud user requests placed in the cloud computing environment for performing mobile computing services involving healthcare data. From the above figure, the scheduling accuracy was found to be neither increasing proportionally nor decreasing proportionally to the samples provided as input. This corroborates the objective that increasing or decreasing the sample size will not affect the output scheduling accuracy. Moreover, the scheduling accuracy using the proposed KHSAROSS method was found to be comparatively better than OO-DRL^1^ and HMHHO^2^. The scheduling accuracy improvement using the proposed KHSAROSS method would contribute to the application of Snow Ablation-based Resource Optimization and Stride Scheduling algorithm. By applying this algorithm first resource optimization was performed using candidate solution quality measured based on the QoS. This, in turn, improved the scheduling accuracy of the proposed KHSAROSS method by 6% compared to^1^ and 17% compared to^2^.

#### Performance analysis of scheduling time

The core function for mobile computing services in the healthcare sector is to map incoming cloud user-requested tasks to the appropriate virtual machines on the basis of factors like processing power, memory needs, and available capacity. To be more specific, scheduling time refers to the procedure of allocating computing resources like memory, bandwidth, and CPU time to different tasks with the objective of optimizing resource utilization by mapping tasks to available virtual machines. Scheduling Time ‘$\:ST$’ is mathematically represented as given below.

21 $$\:ST=\sum\limits_{i=1}^{N}{S}_{i}*Time\:\left[{S}_{SA}\right]$$

From the above Eq. (21), ‘$\:ST$’ is measured based on the samples or the cloud user’s requested tasks in the queue.$\:{S}_{i}$’ and the time consumed in scheduling accurately ‘$\:Time\:\left[{S}_{SA}\right]$’. It is measured in terms of seconds (sec).

**Fig. 7:**
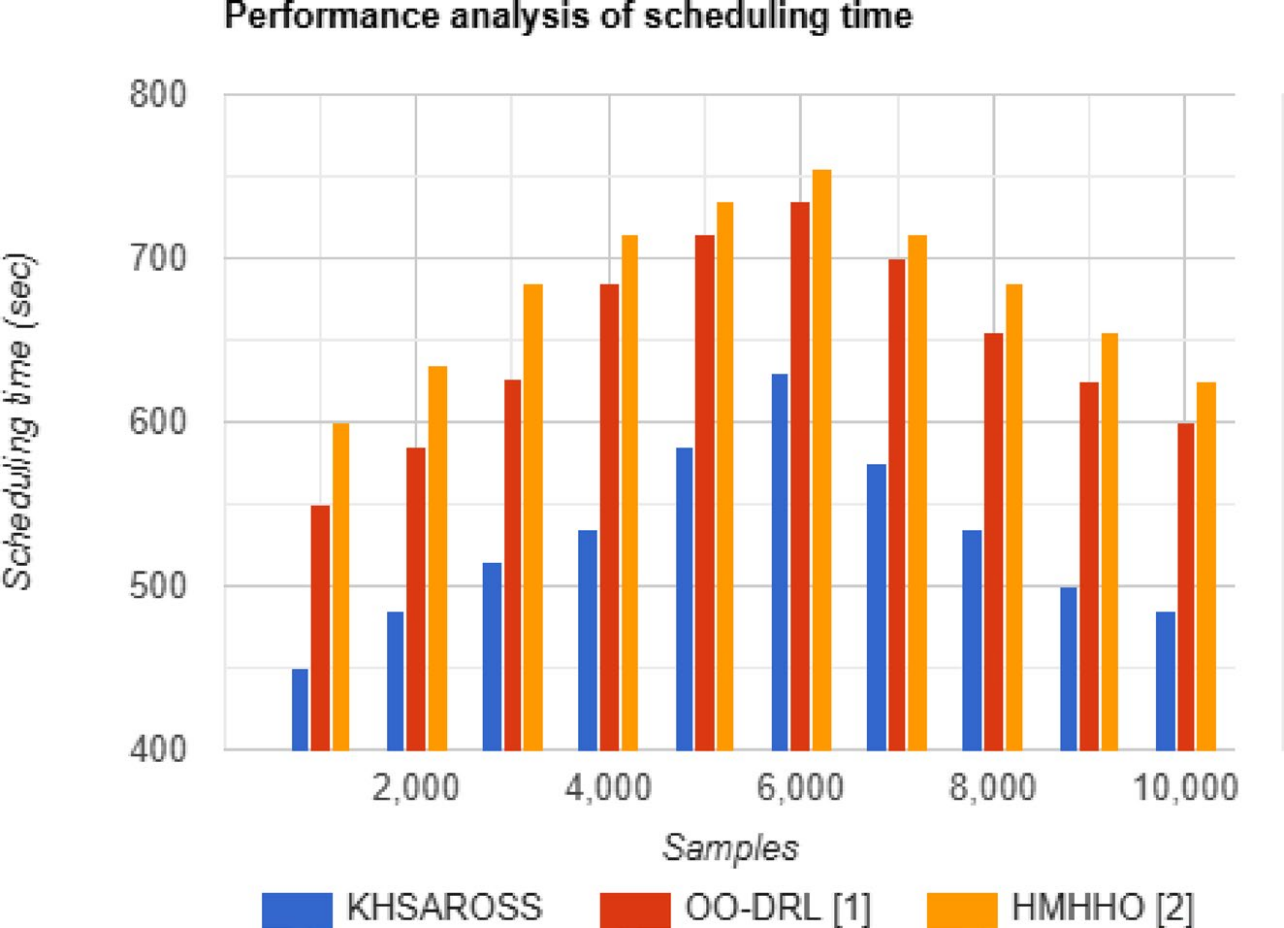
Scheduling time evaluations of existing methods with the proposed method.

Figure 7 given above illustrates the scheduling time measured in terms of seconds (sec) with respect to sample size ranging between 1000 and 10,000. The horizontal axis in the above figure represents the samples and the vertical axis represents the scheduling time involved in performing mobile computing services of resource optimization and scheduling in healthcare sector. From the above figure, the scheduling time using all the three methods are neither said to be increasing or decreasing with the increase in the sample size, therefore corroborating the objective of efficient scheduling in an extensive manner. Moreover, the scheduling time of the KHSAROSS method was found to be comparatively lesser than^1^ and ^2^. The reason was due to the application of Snow Ablation-based Resource Optimization and Stride Scheduling algorithm. By applying this algorithm only after the optimization process of resources being allocated the scheduling process was performed employing Stride Scheduling. By applying this scheduling technique overloaded virtual machine were balanced with that of less loaded virtual machine. This in turn aided in improving the overall scheduling process, therefore reducing the scheduling time using KHSAROSS method by 18% compared to^1^ and 22% compared to^2^.

#### Performance analysis of throughput

In mobile computing services performed via cloud computing, throughput is the amount of data that is transferred across a storage system within a given time period. High throughput means that more data or cloud user requests are said to be transferred in less time and vice versa. It is measured in terms of bits per second (bps).Table 3 tabulates the through rate of three different methods, KHSAROSS, OO-DRL^1^ and HMHHO^2^.

**Table 3:** Throughput evaluation.

S. No	Methods	Throughput (bps)
1	KHSAROSS	1350
2	OO-DRL^1^	1235
3	HMHHO^2^	1015

From the above table results the throughput analysis using the proposed KHSAROSS method was observed to be comparatively better than OO-DRL^1^ and HMHHO^2^. The throughput improvement was owing to the application of QoS fitness function in the exploration phase of the Snow Ablation-based Resource Optimization and Stride Scheduling algorithm and in exploitation stage, virtual machines or healthcare service providers are prompted to employ high-quality solutions adjacent the current best solution. This in turn aids in improving the overall throughput of proposed KHSAROSS method by 9% upon comparison to^1^ and 22% upon comparison to^2^.

#### Computation overhead (CO)

It is measured as an amount of time consumed to generate hash value using Kademlia Hash function for data storage on cloud server.

22 $$\:CO=D*time\left({GH}_{Value}\right)$$

where,$\:\:D$ indicates a data, $\:time\left({GH}_{Value}\right)$represents generating hash value time on cloud server. It is measured in terms of milliseconds (ms).

**Fig. 8:**
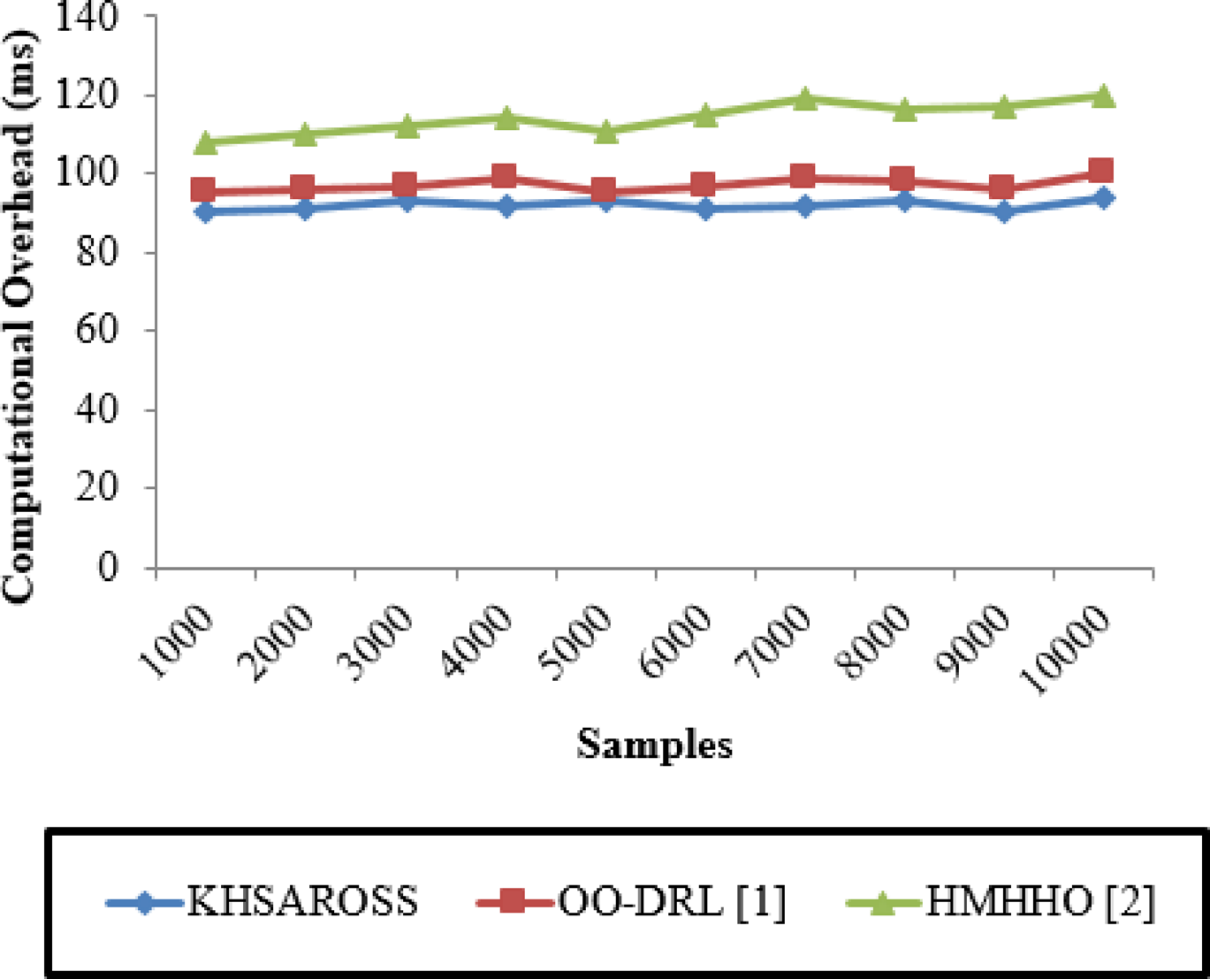
Computational overhead evaluations of existing methods with the proposed method.

Figure 8 given above illustrates the Computational overhead computed in terms of milliseconds (ms) with respect to sample size ranging between 1000 and 10,000. The horizontal axis in the above figure represents the samples and the vertical axis represents the computational overhead involved in performing mobile computing services of resource optimization and scheduling in healthcare sector. Moreover, the computational overhead of the KHSAROSS method is comparatively minimum than^1^ and ^2^. The reason was due to the application of using Kademlia Hash function. This in turn to minimize the computational overhead using KHSAROSS method by 5% compared to^1^ and 19% compared to^2^.

#### Storage complexity (SC)

It is defined as the amount of memory space utilized by data storing on cloud through the hash function and it formulated as follows.

23 $$\:SC=D*memory\:consumption\:\left(DS\right)$$

where, ‘D’ represents a patient data, ‘$\:memory\:consumption\:\left(DS\right)$’ denotes memory space consumed by data storing on cloud server. The storage complexity is measured in terms of Kilobytes (KB).

**Fig. 9:**
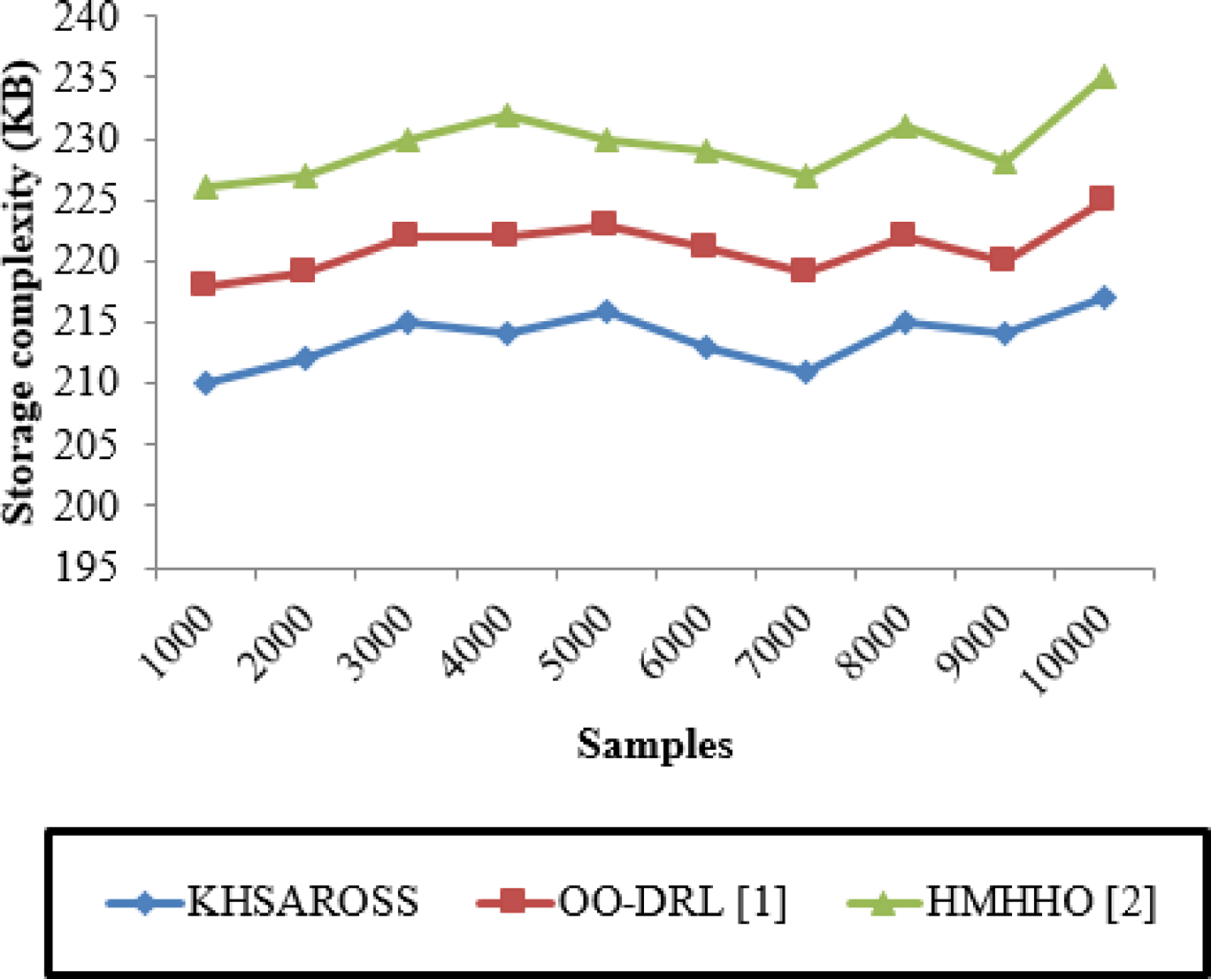
Storage complexity evaluations of existing methods with the proposed method.

Figure 9 given above illustrates the Storage complexity measured computed in terms of kilobytes (KB) with respect to sample size ranging between 1000 and 10,000. In the above figure, horizontal axis represents the samples and the vertical axis represents the memory consumed during mobile computing services of resource optimization and scheduling in healthcare sector. Moreover, the computational overhead of the KHSAROSS method is comparatively lesser than^1^ and ^2^. The reason was due to the application of using Kademlia Hash function. This in turn to decreases the storage complexity using KHSAROSS method by 4% compared to^1^ and 7% compared to^2^.

#### Statistical test

F-test or ANOVA test is a statistical test for computing of two nominal predictor variables on a nonstop result variable. An output ANOVA test of has two independent variables on a dependent variable. ANOVA test outcomes were utilized in an F-test.

**Table 4:** Comparison of ANOVA test for proposed and existing methods.

ANOVA test			
Parameters	OO-DRL^1^	HMHHO^2^	Proposed KHSAROSS
Makespan (sec)	1141.3	1103	796.5
Latency (sec)	956.5	992.5	651
Scheduling accuracy (%)	89.565	81.32	95.115
Scheduling time (sec)	647.6	680.5	529.5
Throughput (bps)	1235	1015	1350
Computational overhead milliseconds (ms)	97.2	114.2	91.9
Storage complexity Kilobytes (KB).	221.1	229.5	213.7

Table 4 shows a comparison of the ANOVA test for proposed and existing methods. The overall obtained result of the proposed KHSAROSS method offers better performance than other state-of-the-art methods.

## Conclusion

Real-time data sharing between health-care providers ensures robust coordination of care plans that in turn aids in healthcare delivery to even underserved populations. A plethora of optimization techniques have been deployed to solve the issue of optimal resource usage and scheduling in mobile computing servicers in healthcare sector. In this work, scheduling process with resource efficient mobile computing services called, KHSAROSS is proposed. To begin, Kademlia Hash Function-based data collection model was applied to the cloud user request schedules to perform efficient data collection with minimal latency and makespan. This eventually leads to a decrease in the makespan and improves resource efficient mobile computing services in healthcare sector. The Snow Ablation-based Resource Optimization and Stride Scheduling algorithm therefore returning resource optimized virtual machine accurately with minimum time. The results demonstrate that the proposed SQEGB method achieves throughput by 15% and scheduling accuracy by 12% than the existing methods. The results of the proposed KHSAROSS method is provided to minimize the makespan by 28%, latency by 32% and scheduling time by 20% compared to the conventional methods.

The limitation of proposed method is ineffective noise reduction during preprocessing and failed to address the patient data security with attack scenario. In future, different preprocessing method will be performed to reduce the noise. Also, the secure and efficient data storing methods will be designed for enhancing the security and enabling the attack detection in healthcare. In addition, the key generation, encryption, decryption and multi-factor authentication will be used to protect sensitive patient information from unauthorized access for early detection of potential attacks on healthcare systems.

## Data Availability

The full code and dataset involved in this work can be found at: https://drive.google.com/drive/folders/1SgY6KrMxhhDBeFQ3ysqLRKBAIeSv54je.
